# Value of segmental myocardial strain by 2-dimensional strain echocardiography for assessment of scar area induced in a rat model of myocardial infarction

**DOI:** 10.1186/1476-7120-10-17

**Published:** 2012-04-03

**Authors:** Shu-sheng Liao, Qin-yun Ruan, Mei-yan Lin, Lei Yan

**Affiliations:** 1 Department of Ultrasound, the First Affiliated Hospital of Fujian Medical University, Fuzhou 350005, China; 2 Department of Ultrasound, the First Affiliated Hospital of Wenzhou Medical College, Wenzhou 325000, China

**Keywords:** Strain, Echocardiography, Myocardial infarction, Rat

## Abstract

**Objectives:**

Two-dimensional strain echocardiography (2DSE) technique has enabled accurate quantification of regional myocardial function. This experimental study was aimed to investigate the value of 2DSE in detection of segmental regional myocardial dysfunction induced by fibrosis following myocardial infarction in a small animal (rat) model.

**Methods:**

A rat model of myocardial infarction was established by ligation of the proximal left anterior descending coronary artery in 17 SD rats. Regional myocardial function was detected by 2DSE at baseline and 4-weeks post-infarction, including end-systolic radial strain and strain rate (SR and SrR) and end-systolic circumferential strain and strain rate (SC and SrC) of each of six segments at papillary level. According to the size of scar found by histologic Masson staining, the optimal cutoff points of parameters for detecting scar area were analyzed and the sensitivity and specificity of every parameter to detect myocardial scar were obtained using ROC.

**Results:**

(1) Comparing with parameters measured at baseline, there were significant decreases in SR, SrR, SC and SrC of each segment at 4 weeks post-infarction, with the worst in the infarct area (32.90 ± 8.79 vs 11.18 ± 3.89, 6.28 ± 1.35 vs 3.18 ± 0.47, -14.46 ± 2.21 vs *-*6.30 ± 2.17 and 4.93 ± 0.95 vs 2.59 ± 1.16, respectively) (all *P *< 0.05). (2)By 4 weeks, the myocardium of infarct area (anteroseptum, anterior and anterolateral) had fibrosis (31.33 ± 9.89, 73.42 ± 13.21 and 13.99 ± 3.24%, respectively) with minimal fibrosis in inferoseptal segment (0.32 ± 0.19%), no fibrosis was found in the inferior and inferolateral segments. (3)Significant negative correlations were found between the size of segmental scar and 2DSE parameters (r-value -0.61 ~ -0.80, all *P *< 0.01) with the strongest correlation in SR. SR less than 10% has 84% sensitivity and 98% specificity for detecting segments of scar area greater than 30% with AUC = 0.97.

**Conclusions:**

2DSE is able to assess regional myocardial dysfunction in a rat model of myocardial infarction and has high accuracy in detecting infarct segments with scar area greater than 30%.

## Grants

National Natural Science Foundation of China(81171360) and Department of Science and Technology of Fujian Province of China (JS09008)

The quantification of regional myocardial function serves as a valuable tool for the diagnosis and monitoring of cardiac function in patients with myocardial infarction and/or heart failure. Rats are widely used as an animal model for investigating cardiovascular disease and there is an increasing demand for noninvasive imaging modalities that are capable of accurately assessing regional myocardial function in small animal models of cardiovascular disease. Two-dimensional strain echocardiography (2DSE) overcomes angle-dependency and provides the capability to measure radial, circumferential and longitudinal components of myocardial motion, and it was recently validated in a variety of settings [1-3]. However, there are few studies that evaluated the application of this technique in small animal models. The goal of this experimental study was to investigate the value of 2DSE in detection of segmental regional myocardial dysfunction induced by fibrosis following myocardial infarction in a rat model.

## Methods

### Experimental protocol

25 Sprague-Dawley rats, male, 10 ~ 12 weeks old, weighing 250 ~ 300 g, were used in this study. Rats were anesthetized with 10% chloral hydrate(4 ml/kg), underwent tracheotomy, intubation and placed on a rodent ventilator. A left thoracotomy was performed to expose the heart. The proximal left anterior descending coronary artery (LAD) was ligated by 6/0 surgical suture. Echocardiography was performed at baseline and 4-weeks after procedures. All animal studies were performed in accordance with the guidelines for the care and use of laboratory animals at our university. The protocol was approved by our local animal protection committee.

### Echocardiography

#### Echocardiographic examination

Echocardiographic studies were performed on VIVID7 dimension'08 (GE Medical). Images were obtained using a 10 S transducer (5.4~11.8 MHz) with high temporal and spatial resolution. The transducer was placed directly on the chest wall and then fixed at the parasternal short-axis view slightly. A two-dimension and M-mode echocardiogram was performed under sedation. Image depth was 1.5 to 2.5 cm, 200 ~ 250 frames/s acquisition, a sector angle of 15 ~ 30 degrees, and with electrocardiographic gating. Images were acquired at baseline and 4-weeks after procedures. Digital data of 3 consecutive heart cycles were recorded and transferred to a personal computer with workstation for offline analysis. The LV was divided into 6 segments at the parastermal short axis view of the mid left ventricle as defined by the American Society of Echocardiography.

#### Image analysis

The images were sent to a workstation with software (EchoPAC 7.0, GE Medical) for analysis. The method has been previously described [4,5]. An assumption was made that the natural acoustic speckles change position from frame to frame in conjunction with the motion of surrounding tissue. The software automatically selected acoustic tissue-tracking regions of interest within the myocardium for tracking and computed the parameters of the 6 segments of the mid left ventricle throughout the cardiac cycle. The parameters including the peak systolic radial strain and radial strain rate (SR and SrR) and peak systolic circumferential strain and circumferential strain rate (SC and SrC) of each of the 6 segments were measured. From the 2-D image of the mid left ventricle, M-mode through the plane of the anterior septal and inferolateral walls was used to measure the left ventricular internal diameter at end diastole (LVIDd) and end systole (LVIDs), and left ventricular end-diastolic (LVEDV) and end-systolic volumes (LVESV) were calculated. Fractional shortening (FS) was calculated using the formula: FS = (LVIDd- LVIDs)/LVIDd × 100%. Left ventricular ejection fraction was calculated by the Teichholtz formula. Three consecutive heart beats were measured and averaged.

### Histology

By 4 weeks, all animals were killed with an overdose of potassium chloride. The hearts were harvested, perfused with 10% formalin under constant pressure for 24 h, and sectioned into three equal divisions perpendicular to the LV long axis. The midventricular segment was paraffin embedded according to established protocols. Tissue sections (5/m thick) were applied to glass slides, which were dried in an oven, deparafinized in xylene, and stained with hematoxylin and eosin and Masson trichrome stains. Photomicrographs of each section stained by Masson trichrome were acquired using a scanner. Segmental infarct size was calculated by dividing the LV cross-sectional area at the papillary muscle level into six segments as described. Infarct size of each segment was estimated by quantification of fibrillar collagen accumulation stained by Masson trichrome using ImagePro Plus (version 5.0) image analysis software. We calculated each segmental infarct size as follows: Infarct size (%) = (segmental collagen area/segmental area) × 100%. It has been previously shown that this type of fibrosis estimation corresponds well with the more quantitative ways of its measurement.

According to previously published [6,7], the mid left ventricle segments were divided into 3 regions: infarct (anteroseptum, anterior and anterolateral), peri-infarct (inferoseptum and inferolateral), and remote (inferior) zones.

### Interobserver and intraobserver variability

8 rats' strain and strain rate data sets were randomly selected from baseline and 4-weeks post-infarction. To determine the interobserver variability for strain and strain rate values, data analysis was repeated by a second observer who was blinded to the values obtained by the first observer. To assess intraobserver variability, the analysis of the data was repeated one week later by the same observer. Interobserver and Intraobserver Variability were measured by calculating the ratio of the mean difference between two measurements over the mean of those measurements.

### Statistical analysis

Data are expressed as mean ± standard deviation. Statistical analysis was performed with SPSS for Windows (Version 11.5). Statistical analysis for comparison of strain and strain rate between animals were performed by repeated-measures analysis of variance. Two-way repeated measures analysis of variance with pairwise multiple comparison procedures (Holm-Sidak method) was used to compare baseline and post-infarct strains and strain rates in different segments. Pearson's correlation was used to describe the relationship between strain, strain rate and infarct size. To assess predictors of segmental strain and strain rate, a forward stepwise multiple linear regression analysis was used with segmental fibrosis and infarct area as possible predictors. The optimal cutoff values of strain and strain rate for detecting myocardial infarction were determined along with sensitivity and specificity using receiver operating characteristic analysis (ROC). A significant *P *value was set at 0.05.

## Results

5 rats died during the experimental protocol and 3 ones died in the following 4 weeks, with a 32% mortality rate totally. Therefore, 17 rats were available for both echocardiographic and histological analysis in this study.

### Two dimensional echocardiography measurements

By 4 weeks, there was a significant increase of left ventricular volumes [*P *< 0.05], and LVEF was significantly reduced following myocardial infarction [(53 ± 8)% vs(82 ± 3)%, *P *< 0.05].(Table 1)

**Table 1:** The parameters of two dimensional echocardiography in rats at baseline and 4 weeks post-infarction

Parameters	4-weeks	Baseline	*P *Value
LVIDd(cm)	0.86 ± 0.04	0.67 ± 0.18	< 0.05

LVIDs(cm)	0.65 ± 0.06	0.38 ± 0.12	< 0.05

LVEDV(ml)	1.37 ± 0.18	0.71 ± 0.11	< 0.05

LVESV(ml)	0.65 ± 0.17	0.15 ± 0.03	< 0.01

LVEF(%)	52.77 ± 8.19	82.34 ± 3.51	< 0.01

FS(%)	24.06 ± 5.07	43.34 ± 2.45	< 0.05

### Strain and strain rate

The changes of values for strain and strain rate of each of the 6 segments are summarized in Table 2. There was a significant reduction in radial strain and radial strain rate (SR and SrR) and circumferential strain and circumferential strain rate (SC and SrC) of all LV segments.

**Table 2:** The parameters of each of 6 segments derived from 2DSE in 17 rats at baseline and 4-weeks post-infarction

Parameters			Infarct area		Peri-infarct area		Remote area
		
		Anteroseptum	Anterior	Anterolateral	Inferoseptum	Inferolateral	Inferior
SR/%^#**▲**^	Baseline	32.29 ± 9.55	32.90 ± 8.79	33.92 ± 10.03	35.27 ± 11.06	37.58 ± 9.35	37.98 ± 9.84
	
	4-weeks	11.60 ± 4.02*	11.18 ± 3.89*	21.85 ± 7.96*	22.75 ± 4.78*	31.72 ± 8.78	31.51 ± 6.55*

SrR/s^-1^	Baseline	6.55 ± 1.18	6.28 ± 1.35	6.72 ± 1.20	7.38 ± 1.70	7.72 ± 1.27	7.79 ± 1.33
	
	4-weeks	3.66 ± 1.01*	3.18 ± 0.47*	4.24 ± 0.77*	4.31 ± 1.51*	4.64 ± 1.06*	4.75 ± 1.33*

SC/%^#**▲**^	Baseline	-14.12 ± 2.94	-14.46 ± 2.21	-12.69 ± 2.44	-14.77 ± 3.43	-11.95 ± 2.55	-14.42 ± 3.15
	
	4-weeks	-7.46 ± 1.39*	-6.30 ± 2.17*	-10.01 ± 2.15*	-11.35 ± 3.16*	-10.61 ± 2.05	-12.97 ± 3.31

SrC/s^-1 #**▲**^	Baseline	4.44 ± 0.63	4.9 ± 0.95	4.19 ± 0.82	4.69 ± 0.88	4.28 ± 0.58	4.79 ± 1.07
	
	4-weeks	3.52 ± 1.08*	2.59 ± 1.16*	3.10 ± 0.67*	3.82 ± 1.21*	3.19 ± 0.36*	3.93 ± 0.54*

*SR*, Radial strain; *SrR*, Radial strain rate; *SC*, Circumferential strain; *SrC*, Circumferential strain rate

*4-weeks vs Baseline, *P *< 0.05; # Infarct area vs Peri-infarct area, *P *< 0.05; ▲Infarct area vs Remote area

### Histological findings *(infarct size)*

**Figure 1:**
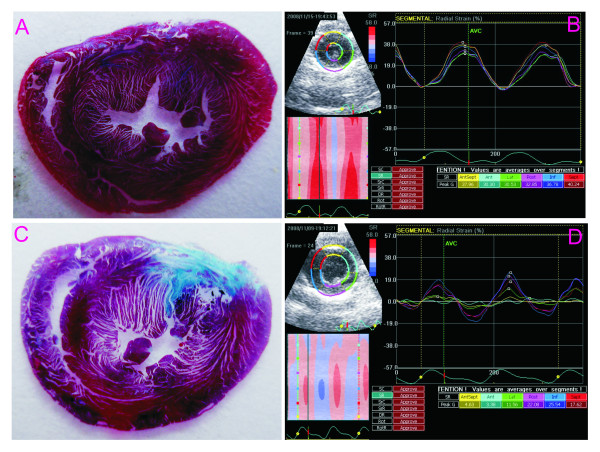
**SR derived from 2DSE and Infarct area in histology**. A: an example of left ventricular cross section of the normal heart stained by Masson trichrome; B: the maps of the peak systolic radial strain of the same heart; C: an example of left ventricular cross section of the heart with myocardial infarct that shows region with replacement fibrosis in blue; D: corresponding segmental peak systolic radial strain that shows significant reduction in the region of infarction.

By 4 weeks, histological analysis demonstrated that the myocardium of infarct area (anteroseptum, anterior and anterolateral) had fibrosis to a variable degree, and for each of these regions the area of fibrosis was 31.33 ± 9.89, 73.42 ± 13.21 and 13.99 ± 3.24% respectively. There was minimal fibrosis in inferoseptal segment (the area was 0.32 ± 0.19%), and, no fibrosis was found in the inferior and inferolateral segments (Figure 1).

### Analysis of 2DSE parameters and infarct size

**Figure 2:**
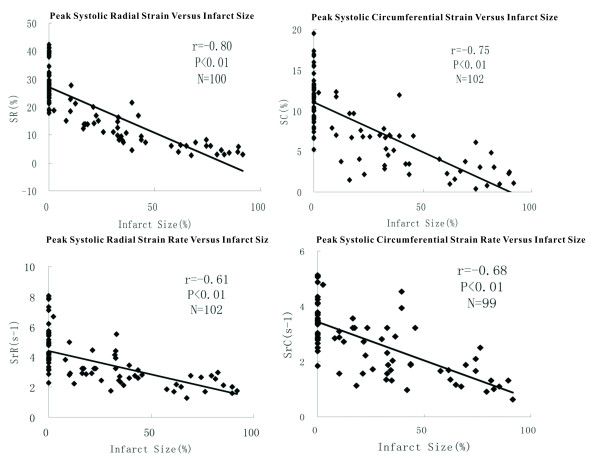
**Scatter plot and Pearson's correlation of 2DSE parameters versus infarct size**.

There was a significantly negative correlations between 2DSE parameters and the area of segmental scar(r = -0.80, -0.61, -0.75 and -0.68 for SR, SrR, SC and SrC, respectively, all *P *< 0.01), with the strongest correlation observed with SR (Figure 2).

**Figure 3:**
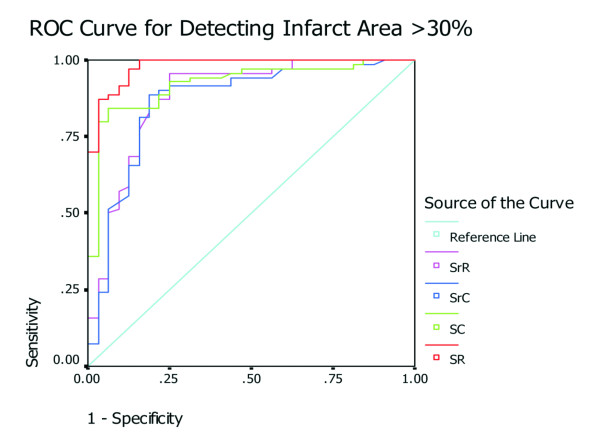
**ROC curve of strain and strain rate to identify segments with greater than 30% area of infarction**.

According to the size of scar found by histology, the receiver operating characteristic curve analysis showed that SR had the highest accuracy in detecting segmental myocardial infarction. SR less than 10% had 84% sensitivity and 98% specificity for detecting segments of scar area greater than 30%. Moreover, the area under the curve (AUC) for SR was the highest with 0.97, compared with SC, SrC, and SrR (AUC = 0.92, 0.87 and 0.87, respectively) (Figure 3).

Reproducibility. The intraobserver and interobserver variabilities for the different 2-dimensional strain and strain rate measurements are listed in Table 3. The largest difference was observed for circumferential strain rate.

**Table 3:** Variabilities of Myocardial Strain and Strain Rate Measurement by 2-Dimensional Strain

	Radial strain	Circumferential strain	Radial strain rate	Circumferential strain rate
Intraobserver	5.8%	7.6%	6.3%	8.5%

Interobserver	6.1%	6.9%	8.0%	11%

## Discussion

Rats are widely used as an animal model for investigating cardiovascular disease, such as heart failure, acute myocardial infarction, and hypertension [8-10]. Many studies have used M-mode and 2D echocardiography for the assessment of ventricular function in rats [11,12]. Although these technique are adequate in the absence of regional wall-motion abnormalities, the small size of the rat heart and the relatively fast heart rate preclude accurate measurements in case of regional wall-motion abnormalities. Doppler-derived strain measurements is a valuable technique for the quantification of regional myocardial contractility that has been validated in a variety of experimental animal models and clinical settings [13-15]. However, technical issues, including angle dependence, signal noise, and measurement variability, may limit its routine use in rat models.

Recently, two dimensional strain echocardiography, which is based on speckle tracking, has been validated in the LV with sonomicrometry and magnetic resonance imaging tagging in vitro and in vivo, and new clinical applications of this technique on LV function are currently being explored [16-18]. In contrast to TDI derived parameters, speckle tracking is an angle independent technique as the movement of speckles can be followed in any direction. The major advantage of this new imaging technique is that it enables analysis of radial and circumferential strain and strain rate from short-axis views. Thus, we focused on analysis of the new available parameters. The aim of this study was to assess sensitivity of radial and circumferential strains to detect infarct-induced left ventricular (LV) scarring in a rat model. As expected, the study demonstrated that 2DSE was useful in identifying infarcted myocardium versus noninfarcted myocardium, as determined by histology. SR less than 10% had 84% sensitivity and 98% specificity for detecting segments of scar area greater than 30%.

It is now understood that the regional cardiac performance changes earlier than the global function. Reports from several settings showed that myocardial strain and strain rate have the potential to discriminate viable from infarcted myocardium [5,19]. After MI, the velocity of cardiac contractility decreases, which is reflected by the reduction of strain. Derumeaux et al. [20] have found that Doppler myocardial imaging could differentiate nontransmural from transmural infarction in open-chest pigs study. In this study we found that SR, SC, SrR and SrC in the LAD territory regions were markedly reduced at 4 weeks post-MI. Radial strain relates to the extent of myocardial wall thickening, whereas radial strain rate relates to the velocity of wall thickening. Circumferential strain and strain rate relate to fiber shortening along a circumferential line. Generally, longitudinal strain measurements are more robust than radial measurements, because the apical window allows interrogation of all LV myocardial segments. There were some reports that showed 2DSE to work better for measurements of deformation in the longitudinal direction than in other directions in humans and large animals [19,21]. A recent study reported that longitudinal strain after primary reperfusion therapy is an excellent predictor of LV remodeling and adverse events in patients with anterior wall acute myocardial infarction [22]. Another research concluded that the combined assessment of both long- and short-axis function using 2D strain may be useful to identify the transmural extent of myocardial infarction [23]. Because of the suboptimal 2D image quality from the apical window in a rat heart, only radial and circumferential strains from the short axis views were assessed in our study. By 4 weeks, the histological analysis showed that the myocardium of infarct area had fibrosis, albeit to a variable degree, and there was a significant reduction in both radial and circumferential strain. Becker et al. [24] reported that the peak systolic radial and circumferential strain and strain rate enable the distinction between normal, hypokinetic and akinetic segments as defined by magnetic resonance imaging(MRI), and compared with systolic radial strain rate, systolic radial strain enable better the distinction between different functional states defined by MRI. Our results also showed that radial strain had the highest accuracy in detecting segmental myocardial infarction.

Adverse left ventricular remodeling occurs within hours following infarction and is one of the most important prognosticators for short and long term survival. Heart failure is a result of progressive cardiac enlargement and dysfunction, processes that continue months to years after the event. The two fundamental processes that occur after a large anterior MI and that are responsible for the occurrence of heart failure, arrhythmias and death are replacement fibrosis and LV dilation through adverse remodeling. In theory, replacement fibrosis affects strain through loss of contractile elements, whereas remodeling affects cardiac function through increased stress and decreased contractility of remaining myocardium. Our results indicate that in post myocardial infarct LV remodeling, the parameters of 2DSE can characterize both the functional and pathologic changes in the underlying tissue. These findings are consistent with the previous reports that peak-systolic and early diastolic strain rates, which closely correlate with strain, show strong dependence on the development of myocardial fibrosis [7]. The assessment of alterations of regional myocardial function and LV remodeling after myocardial infarction is very important for prognosis and therapy, including revascularization and stem cell therapy. In evolving infarcts, myocardial fibrosis is increased along with LV remodeling, and reduced tethering between myofiber bundles, leading to impaired cardiac function [25]. Our results show that there are significant correlations between strain/train rate and the extent of fibrosis. So, 2DSE can be utilized as a noninvasive tool for monitoring changes in regional function after therapeutic interventions, such as stem cell transplantation after myocardial infarction.

Interestingly, we found that the strain and strain rate were also decreased in the regions without or with minimal infarction. This is consistent with previous reports demonstrating dysfunction in adjacent and remote noninfarcted regions after anterior myocardial infarction in both animal and human subjects [6,7,26]. Although the mechanisms underlying this dysfunction were not well elucidated, the increased regional wall stress and systolic myocardial stiffness, which are due to severe edema, cellular and hemorrhagic infiltration, and depressed coronary flow reserve may be the possible contributors [27,28]. Previous studies have shown that following infarction ventricular remodeling occurs not only because of myocyte loss in the infarct region, but also due to a myopathic process in the peri-infarct border zones, and the apoptosis may play a role as well [29].

Limitations. There are some limitations to this study. Firstly, strain is a three-dimensional tensor, whereas our measurements were only in a two-dimensional plane, because of the small size of the rat heart, we were not able to acquire adequate apical views and thus could not assess longitudinal strain. Recently developed 3D speckle tracking techniques may result in a higher feasibility for measuring longitudinal strain [30,31]. Secondly, in our method, coregistration between echocardiographic images and histology slices is important, as errors can occur if segments were not matched. So, we used the LV two papillary muscles and the two fixed anatomic landmarks, the anterior and posterior insertion of the right ventricle to the LV, to match segments between 2D images and histology sections [32]. Finally, we did not acquire hemodynamic measurements in these animals which are known to affect cardiac function.

In conclusion, 2DSE can identify segmental LV dysfunction induced by fibrosis following myocardial infarction in a rat model. Regional strain and strain rate are impaired after MI, possibly as a result of diminished myocardial contractility and increased fibrosis. The persistent myocardial dysfunction in the peri-infarct and remote regions together with the dysfunction in the infarct region were associated with progression of left ventricular enlargement and adverse remodeling. 2DSE appears well suited for the assessment of regional function post-MI.

## Competing interests

The authors declare that they have no competing interests.

## Authors' contributions

QR designed the research; QR and SL performed the research, ML and LY participated in echocardiographic image analysis; QR and SL analyzed the data and wrote the manuscript. All authors read and approved the final manuscript.
